# Low physical activity is associated with two hypokinetic motor abnormalities in psychosis

**DOI:** 10.1016/j.jpsychires.2021.11.014

**Published:** 2021-11-08

**Authors:** Sebastian Walther, Irena Vladimirova, Danai Alexaki, Lea Schäppi, Kathrine S. F. Damme, Vijay A. Mittal, Stewart A. Shankman, Katharina Stegmayer

**Affiliations:** a Translational Research Center, University Hospital of Psychiatry and Psychotherapy, University of Bern, Switzerland; b Department of Psychology, Northwestern University, Evanston, IL, USA; c Department of Psychiatry and Behavioral Sciences, Northwestern University, Chicago, IL, USA

**Keywords:** Actigraphy, Schizophrenia, Catatonia, Cardiometabolic health

## Abstract

Individuals with schizophrenia engage in more sedentary behavior than healthy controls, which is thought to contribute to multiple health adversities. Age, medication side effects and environment are critical determinants of physical activity in psychosis. While motor abnormalities are frequently observed in psychosis, their association with low physical activity has received little interest. Here, we aimed to explore the association of actigraphy as an objective measure of physical activity with clinician assessed hypokinetic movement disorders such as parkinsonism and catatonia. Furthermore, we studied whether patients with current catatonia would differ on motor rating scales and actigraphy from patients without catatonia. In 52 patients with schizophrenia spectrum disorders, we cross-sectionally assessed physical activity using wrist actigraphy and ratings of catatonia, parkinsonism, and negative syndrome. The sample was enriched with subjects with severe psychomotor slowing. Lower activity levels correlated with increased age and severity of catatonia and parkinsonism. The 22 patients with catatonia had lower activity as well as higher scores on parkinsonism, involuntary movements, and negative symptoms compared to the 30 patients without catatonia. Collectively, these results suggest that various hypokinetic motor abnormalities are linked to lower physical activity. Therefore, future research should determine the direction of the associations between hypokinetic motor abnormalities and physical activity using longitudinal assessments and interventional trials.

## 1. Introduction

Cardio metabolic health is a major issue in schizophrenia spectrum disorders. Schizophrenia spectrum disorders are associated with lower life expectancy of 15–20 years, which may be partially accounted for the impact of these disorders on health behaviors (Hjorthoj et al., 2017; Kurdyak et al., 2021; Nielsen et al., 2021). Meta-analyses indicate that subjects with schizophrenia have lower physical activity (PA), particularly of moderate to vigorous activity than healthy controls (moderate activity – 10 min per day, vigorous activity – 3 min per day) (Stubbs et al., 2016; Vancampfort et al., 2017). Some of the studies on PA in schizophrenia suggested a negative impact of side effects of antipsychotic medication (Vancampfort et al., 2017; Vancampfort et al., 2012), e.g. fatigue or metabolic syndrome. Although, low PA and low fitness have also been observed in unmedicated subjects at risk for psychosis (Damme et al., 2021; Mittal et al., 2013). In addition, motor side effects may also contribute to lower PA in subjects with schizophrenia.

A variety of spontaneous and drug induced motor abnormalities are frequently observed in schizophrenia spectrum disorders (Walther and Mittal, 2017; Walther et al., 2020). Both hyperkinetic movement disorders, such as abnormal involuntary movements, akathisia or dystonia, and hypokinetic movement disorders, e.g. Parkinsonism (with brady-kinesia), or catatonia may occur at all stages of the disorder ranging from those at risk to those with chronic courses (Peralta and Cuesta, 2010, 2017; Walther and Mittal, 2017; Walther and Strik, 2012; Walther et al., 2020). Finally, psychomotor slowing remains a special symptom, as it may occur either independently or concurrently with classic hypokinetic motor abnormalities or the negative syndrome domain of apathy (Mittal et al., 2021a).

Assessing motor abnormalities, particularly the hypokinetic forms, remains challenging in clinical practice. In fact, there is considerable overlap between hypokinetic phenomena, such as catatonia, parkinsonism, the negative syndrome and psychomotor slowing in psychosis (Mittal et al., 2021a; Osborne et al., 2020; Rogers, 1985; Walther and Mittal, 2017). Depending on the perspective, the same hypokinetic behavior will receive different names. Essentially, these overlaps will also confound expert rating scales, that are typically designed to focus on a single type of motor abnormality. Therefore, the use of instrumental assessments has been advocated in schizophrenia (Pieters et al., 2021; van Harten et al., 2017; Walther et al., 2020). Instrumental measures, such as actigraphy, can assess movements across multiple real world settings continuously and allow for generating dimensional objective parameters. Furthermore, instrumental measures are not prone to observer biases and require little training.

One study indicated that lower PA was predicted by parkinsonism and age in patients with schizophrenia (Pieters et al., 2021). However, the association of PA with other hypokinetic movement disorders such as catatonia remains unclear. In contrast, a number of previous studies using wrist-actigraphy consistently reported reduced activity levels to correlate with increased severity of the negative syndrome (Docx et al., 2013; Kluge et al., 2018; Servaas et al., 2019; Walther et al., 2014; Wichniak et al., 2011). Thus, we may assume that lower PA is indeed linked to negative symptom severity. Still, given the conceptual overlap of the expert rating scales, it is currently unclear if objectively assessed PA may be associated with the presence of hypokinetic movement abnormalities, such as catatonia or parkinsonism. Finally, although patients with catatonic schizophrenia have been found to exhibit lower activity levels compared to patients with paranoid schizophrenia in DSM-IV (Walther et al., 2009), it will still be important to interrogate whether patients with and without catatonia differ in objective measures of motor activity as well as in common ratings of hypokinetic movement disorders.

In this study on hypokinetic movement disorders in psychosis, the first aim was to examine correlations between physical activity (PA) as measured by actigraphy with current severity of parkinsonism, catatonia, and negative symptoms. Here, we expected to find strong associations between expert ratings of motor abnormalities and actigraphic measures of PA. The next goal was to determine patients with and without current catatonia exhibited differences on PA and the severity of motor abnormalities. We hypothesized that patients with catatonia will have lower activity levels as well as higher scores on parkinsonism and negative symptoms compared to patients without catatonia.

## 2. Methods

### 2.1. Participants

For this analysis, we combined data from two studies; baseline data of a randomized controlled trial (n = 21) (Walther et al., 2020) and a prior cross-sectional study on motor function and neuroimaging in schizophrenia (n = 31) (Walther et al., 2017). We included all available data sets with (1) actigraphy data and (2) patients with schizophrenia (n = 37), schizophreniform (n = 4) or schizoaffective disorder (n = 3). In total, we included33 men and 19 women (mean age 36.8 ± 12.3 years, mean duration of illness 11.6 ± 11.5 years). All but three patients were on antipsychotic medication at the time of assessment. Thirty-two patients were on monotherapy and 17 received multiple antipsychotics. Current antipsychotics administered included risperidone (n = 14), clozapine (n = 15), olanzapine (n = 11), quetiapine (n = 6), haloperidol (n = 5), aripiprazole (n = 4), amisulpride (n = 3), paliperidone (n = 3), clotiapine (n = 2), zuclopenthixol (n = 2), flupentixol (n = 1), and lurasidone (n = 1). Two of the patients (4%) received antiparkinsonian medication. All subjects were right-handed and diagnoses were established using DSM-5 criteria and the Mini International Neuropsychiatric Interview (MINI) (Sheehan et al., 1998). Subjects in the interventional study were included if they had severe psychomotor slowing (52% of the subjects screened for the trial had severe psychomotor slowing), while the other study included patients irrespective of their current symptom presentation. Common exclusion criteria were neurological or medical conditions that impact motor behavior, e.g. stroke, lifetime diagnosis of substance dependence other than nicotine. All participants were in patients (n = 44) or outpatients (n = 8) at the University Hospital of Psychiatry and Psychotherapy in Bern, Switzerland. The local ethics committee had approved both studies, participants provided written informed consent prior to inclusion.

### 2.2. Procedures

#### 2.2.1. Instrumental measure of PA

Both studies applied identical actigraphy assessments using wrist-worn actigraphs (Actiwatch, Cambridge Neurotechnology, Inc., Cambridge, UK) for 24 consecutive hours on the non-dominant arm. The device contains an accelerometer that converts movements in all directions into movement counts. Data were sampled in 2 s intervals. Participants provided information on recording pauses (due to showering or bathing) and sleep. Only the data collected during wakeful periods of the 24 h recording time were analyzed. The total sum of the movement counts were averaged to provide the activity level (AL) in counts/h as the instrumental measure of physical activity (PA) (Kluge et al., 2018; Walther et al., 2009; Walther et al., 2014). Thus, AL represents the total sum of movements per hour during the wake periods of the day across all types of activity, e.g. sitting, standing, or walking.

#### 2.2.2. Rating scales

Assessments of psychopathology and motor behavior were conducted blind to the outcome of the actigraphy data. All clinical ratings were performed by psychiatry residents (IV, DA, LS, KS), who had been trained by the principal investigator (SW) to achieve κ > 0.80.

##### 2.2.2.1. Motor abnormalities.

Hypokinetic motor abnormalities were assessed with the Bush Francis Catatonia Rating Scale (BFCRS) (Bush et al., 1996) and the Unified Parkinson’s Disease Rating Scale (UPDRS) (Fahn et al., 1987), from which we rated the pure motor part (part III). In one study, we also assessed dyskinesia with the abnormal involuntary movement scale (AIMS) (Guy, 1976) and neurological soft signs with the Neurological Evaluation Scale (NES) (Buchanan and Heinrichs, 1989).

##### 2.2.2.2. Symptom rating scales.

We measured broad symptom severity with the Positive And Negative Syndrome Scale (PANSS) (Kay et al., 1987). Furthermore, negative syndrome severity was rated using the Scale for the Assessment of Negative Symptoms (SANS) (Andreasen, 1989).

### 2.3. Statistics

Shapiro-Wilk tests indicated that none of the variables followed a normal distribution. Therefore, all analyses were conducted with nonparametric tests (Spearman rank correlations and Mann-Whitney-U-test). First, we correlated the activity levels (AL) with PANSS scores. Second, we calculated the correlations between AL, age, CPZ, and the hypokinetic motor behavior scales (BFRCS, UPDRS-III) and SANS. These correlations were corrected for multiple comparisons using false discovery rate (FDR). Third, we tested whether patients with catatonia according to the BFCRS criteria (≥ 2 items on the screening instrument of BFCRS), would differ from those without catatonia (<2 items on the BFCRS screening instrument) on motor behavior rating scales and activity levels using Mann-Whitney-U-tests, and ANCOVAs controlling for age and CPZ. Finally, we correlated activity levels with the three BFCRS factors described by Wilson and colleagues in a sample of 339 patients: decreased, increased and abnormal psychomotor activity (Wilson et al., 2015).

## 3. Results

### 3.1. Correlation of PA with psychopathology

In this sample, the activity levels were on average 14′470 counts/h (SD = 8′136), which is in line with previous reports and much lower than in healthy controls, e.g. mean = 21′511, SD = 7′580, n = 46 in (Walther et al., 2017). Lower activity levels in patients correlated with higher PANSS negative scores (r_S_ = − 0.32, p = .021), but not with PANSS positive (r_S_ = 0.20, p = .15), general (r_S_ = − 0.19, p = .18) or total scores (r_S_ = − 0.20, p = .16). In the subgroup of patients with AIMS and NES scores (n = 31), we found no correlation with activity levels (all p > .39).

### 3.2. Correlation of PA with hypokinetic motor abnormalities

**Fig. 1.**
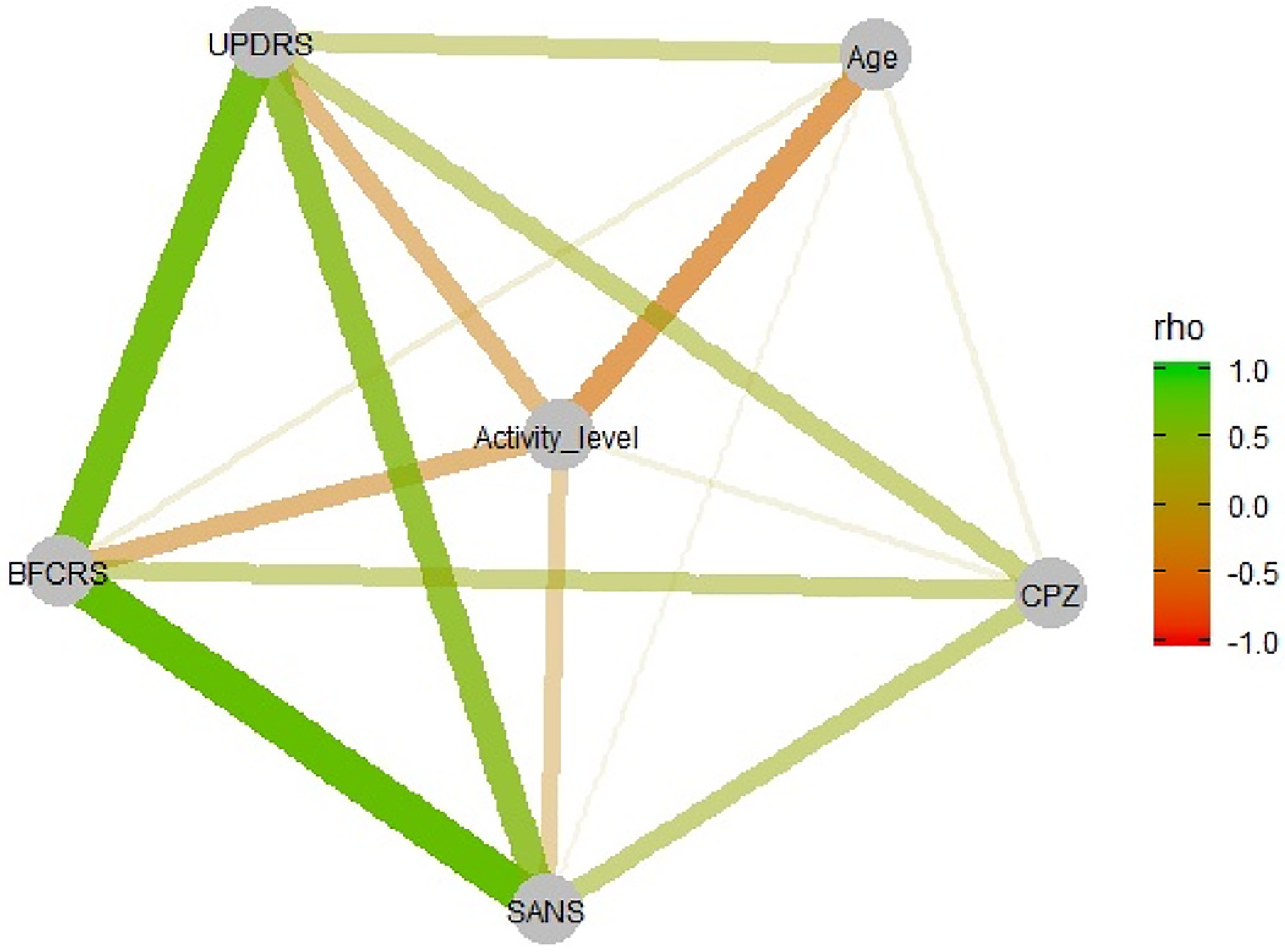
Correlation matrix of physical activity, motor rating scales, negative symptom ratings, age, and antipsychotic medication in CPZ equivalent.

Lower PA was linked to higher age and higher scores on BFCRS, UPDRS, and SANS (see Table 1, Fig. 1). The three rating scales further correlated substantially with each other. Current dose of antipsychotics was unrelated to PA.

### 3.3. Catatonia vs. non-catatonia patients

According to the BFCRS screening instrument, 22 patients qualified for catatonia (13 men and 9 women), while 30 had no catatonia (20 men and 10 women). Groups did not differ in sex (Chi^2^ = 0.31, df = 1, p = .77). Patients with catatonia had lower activity levels, more negative symptoms and higher scores on Parkinsonism and abnormal involuntary movements (see Table 2). Note that 14 of the 22 patients with catatonia also qualified for DSM-5 catatonia criteria.

### 3.4. Correlation of catatonia factors with PA

Activity levels correlated with the BFCRS factor decreased (r_S_ = − .31, p = .025), but not with the factors increased or abnormal (r < −0.21, p > .14).

## 4. Discussion

Physical activity (PA) is critical for health related outcomes, but lower in patients with schizophrenia than in healthy controls. Age, body mass index, and medication side effects are potential contributors to lower PA in subjects with severe mental illness. However, the association with other potential factors such as motor abnormalities on PA have not been investigated so far. This study applied an instrumental measure of PA, i.e. wrist actigraphy, in 52 patients to explore cross-sectional correlations between activity levels (AL) and the severity of hypokinetic motor abnormalities. We found that age, catatonia, and Parkinsonism are associated with low activity levels in schizophrenia spectrum disorders. In addition, patients with current catatonia had lower PA and higher ratings of negative symptoms, Parkinsonism, and abnormal involuntary movements compared to patients without catatonia.

The correlation of low AL with ratings of parkinsonism and catatonia could reflect several underlying issues in psychosis. The most likely explanation is that parkinsonism and catatonia both exert effects on the motor system of patients resulting in reduced PA. Reduced neural output from the basal ganglia and the primary motor cortex in patients with psychosis and catatonia or Parkinsonism has been suggested by neuroimaging studies (Hirjak et al., 2020; Viher et al., 2020; Walther et al., 2017; Wasserthal et al., 2020; Wolf et al., 2021). This reduced output will affect all motor behaviors and thus influence measures of PA. Another possibility could be an indirect effect, i.e. low PA contributes to or exacerbates a variety of motor abnormalities, including parkinsonism and catatonia. We may speculate that reduced motor output resulting from pathological motor circuit activity may be further deteriorated by sedentary behaviors through a lack of training of this circuit. Indeed, in idiopathic Parkinson’s disease physical exercise helps improving motor outcomes, i.e. reducing bradykinesia (Tiihonen et al., 2021). Similarly, anecdotal reports in chronic catatonia suggest beneficial effects of physical exercise (Heckers and Walther, 2021). A third option, which is not mutually exclusive, is supported in part by the fact that correlations were moderate, is that these phenomena may result from multiple partially overlapping but also distinct pathophysiological mechanisms (Mittal et al., 2021a). Finally, a number of clinical parameters such as dosage of current medication or antipsychotic substance used could exert negative effects on both PA and motor abnormalities (Walther et al., 2010). However, while current CPZ were moderately correlated with parkinsonism, catatonia, and negative symptom severity, CPZ was unrelated to our objective measure of PA.

These findings extend previous knowledge by demonstrating that multiple hypokinetic movement disorders, i.e. catatonia and Parkinsonism, may contribute to lower PA in schizophrenia. The result corroborates the only other study applying actigraphy and ratings of Parkinsonism (Pieters et al., 2021). Findings are similar even though the accelerometer placement differed, i.e. hip vs. wrist of the non-dominant arm. While replicating findings on the role of Parkinsonism in PA, this study is the first to correlate the severity of catatonia according to the BFCRS with activity levels measured via actigraphy. Because of its frequency of 8% in clinical populations (Solmi et al., 2018), it is important to consider catatonia as a transdiagnostic motor abnormality. Catatonia may present with increased, decreased, or abnormal psychomotor activity (Heckers and Walther, 2021; Hirjak et al., 2020; Walther et al., 2019; Wilson et al., 2015). Our data demonstrate that lower activity levels correlate with the score on the BFCRS factor decreased activity. While the BFCRS total score was associated with lower PA as hypothesized, single items indicating increased psychomotor activity (e. g. excitement, combativeness) could correlate with higher PA in other samples. More often, however, catatonia presents with decreased psychomotor activity (Wilson et al., 2015), affecting physical activity in the same way as age or Parkinsonism.

Our finding of reduced activity levels in patients with current catatonia is in line with a previous report of our group that compared patients with catatonic schizophrenia to patients with paranoid schizophrenia using wrist actigraphy (Walther et al., 2009). Moreover, the current study corroborates previous actigraphy studies reporting reduced activity levels in schizophrenia patients or subjects at clinical high risk (CHR) with pronounced negative symptoms (Damme et al., 2021; Docx et al., 2013; Kluge et al., 2018; Mittal et al., 2013; Walther et al., 2009; Walther et al., 2014). Finally, our results align with studies reporting moderate to strong correlation between hypokinetic motor abnormalities, e.g. parkinsonism and catatonia, parkinsonism and negative symptoms, catatonia and negative symptoms (Docx et al., 2012; McKenna et al., 1988; Peralta et al., 2012; Rogers, 1985; Sambataro et al., 2020). Still, the correlation was only observed for the PANSS negative syndrome scale, but not for SANS total.

Some of this correlation is likely to stem from conceptual overlap, as all current rating scales have been developed within specific frameworks and focus on one motor phenomenon or on negative symptoms. However, on a behavioral or neurophysiological level the differentiation between the hypokinetic phenomena is close to impossible; therefore, the nature of extrapyramidal signs, catatonia, and negative symptoms such as avolition remains a major challenge to the field (Mittal et al., 2021a,b). Instrumental assessment of motor behavior holds promise in measuring motor behavior precisely (van Harten et al., 2017). However, a revision of current concepts is still needed. This study demonstrates that objectively assessed low physical activity in schizophrenia correlates with all hypokinetic phenomena, rendering the link between low physical activity and negative syndrome scores less specific as previously thought.

The etiology of sedentary behaviors or low PA is still subject of investigations. Future longitudinal studies need to address heterogeneity, i.e. few patients present with pure forms of hypokinetic motor abnormalities, as these behaviors overlap substantially. Most motor phenomena manifest for longer periods of time, may wax and wane, but have the potential to impact physical activity of subjects in addition to unspecific effects of age and environmental enrichment (Mittal et al., 2021a). Another way to make progress would be the revision of the current concepts of motor abnormalities. This would also aid the interpretation of the rising number of excellent neuroimaging studies in the field that have provided first preliminary insight but struggle to translate pathology to specific motor behaviors (Hirjak et al., 2020; Mittal et al., 2021a; Northoff et al., 2021; Walther et al., 2017; Walther et al., 2017; Wolf et al., 2021). Finally, if we achieve a better understanding of the pathobiology of the various motor abnormalities, we may improve efforts to test novel treatments. Future clinical trials will need to establish whether hypokinetic motor abnormalities will be ameliorated with physical exercise interventions increasing PA or whether PA can be increased by treating hypokinetic motor abnormalities with brain stimulation. Indeed, first studies are targeting motor abnormalities with non-invasive brain stimulation with encouraging effects that require replication and extension (Gupta et al., 2018; Lefebvre et al., 2020; Walther et al., 2020; Walther et al., 2020). If some of these novel treatments prove to increase PA, we would also expect benefits for general health outcomes in subjects with schizophrenia.

The strength of this study include the objective assessment of PA and careful clinical assessment of motor abnormalities. Limitations include the lack of longitudinal data, the moderate sample size, and the enrichment of the sample with subjects with severe psychomotor slowing, which may limit the generalizability to all subjects with schizophrenia. Still, psychomotor slowing is frequently observed in this disorder. Importantly, the current dose of antipsychotics was unrelated to PA, but studies in unmedicated first episode patients would add important information on this issue. In an ideal study, ratings of catatonia and parkinsonism would have been conducted independently, i.e. by separate raters blind to the other’s evaluation. Future studies will include longitudinal assessments to study the course of hypokinetic motor abnormalities as well as further objective measures of PA, such as automated gait analyses, which may help to disentangle parkinsonism from catatonia in psychosis. Finally, clinical trials with physical exercise and brain stimulation will shed light on the mechanism between PA and hypokinetic movement disorders.

In sum, this study demonstrates that low PA in schizophrenia is linked to age, negative symptoms, and hypokinetic motor abnormalities such as catatonia and parkinsonism. Future efforts will include multiple objective assessments of motor behavior in longitudinal studies to disentangle the contributions of various motor abnormalities frequently seen in psychosis.

## 5. Contributors

Dr. Walther designed the study, wrote the protocol, acquired funding, supervised data acquisition, analyzed the data and wrote the first draft of the manuscript. Drs. Vladimirova, Alexaki, Schäppi and Stegmayer recruited subjects and conducted assessments. All authors discussed findings and edited the manuscript.

## Figures and Tables

**Table 1:** Correlation of physical activity and clinical parameters in 52 patients with SSD.

	Age	CPZ	BFCRS	UPDRS III	SANS total
**Activity level**	−.45 (.003)	.05 (n.s.)	−.34 (.037)	−.33 (.037)	−.23 (n.s.)
**Age**	–	−.04 (n.s.)	.07 (n.s.)	.28 (n.s.)	−.03 (n.s.)
**CPZ**		–	.31 (.039)	.33 (.037)	.34 (.037)
**BFCRS**			–	.66 (<.001)	.72 (<.001)
**UPDRS III**				–	.55 (<.001)

Spearman rank correlations, p-values are FDR corrected. CPZ – chlorpromazine equivalents, BFCRS – Bush Francis Catatonia Rating Scale, SANS - Scale for the Assessment of Negative Symptoms, UPDRS III – motor part of the Unified Parkinson’s Disease Rating Scale.

**Table 2:** Comparison between patients with and patients without current catatonia.

	Catatonia (n = 22)		No catatonia (n = 30)		Non-parametric statistic		ANCOVA correcting for age and CPZ	
	M	SD	M	SD	U	p	F	p
Age (y)	38.6	12.5	35.3	12.0	275	.313	–	–
CPZ (mg)	623.6	436.5	344.6	340.2	181	.005	–	–
Activity level (counts/h)	10′ 866.0	5′ 144.6	17′ 113.3	8′ 953.3	170	.003^a^	6.0	.002
UPDRS III	15.9	8.4	5.8	5.4	88	<.001^a^	11.7	<.001
AIMS	8.8	7.3	1.0	1.5	7	<.001^a^	12.4	<.001
NES	13.5	13.4	12.2	11.8	74	.990	.6	.612
SANS	73.2	27.2	30.8	21.4	76	<.001^a^	13.1	<.001
PANSS pos	15.6	6.7	17.9	5.9	249	.137	.9	.435
PANSS neg	31.6	8.4	18.6	6.3	70	<.001^a^	13.5	<.001
PANSS total	94.8	22.2	71.2	16.3	144	.001^a^	7.3	<.001

a Indicates group differences that survive Bonferroni correction, AIMS – abnormal involuntary movement scale, CPZ – chlorpromazine equivalents, NES – neurological evaluation Scale, PANSS – positive and negative syndrome scales (positive, negative and total scores), SANS – scale for the assessment of negative symptoms, UPDRS III – motor part of the Unified Parkinson’s Disease Rating Scale.
